# 9,9′-Dibromo-9,9′-bifluorene

**DOI:** 10.1107/S160053680800295X

**Published:** 2008-02-06

**Authors:** Yoshiyuki Mizuhata, Norihiro Tokitoh

**Affiliations:** a Institute for Chemical Research, Kyoto University, Gokasho, Uji, Kyoto 611-0011, Japan

## Abstract

9,9′-Dibromo-9,9′-bifluorene, C_26_H_16_Br_2_, has a *gauche* conformation about the connecting C—C bond [the Br—C—C—Br torsion angle is 59.39 (16)°]. The crystal structure is sustained mainly by an inter­molecular C—Br⋯π inter­action [3.299 (2) and 3.369 (2) Å] towards the bifluorene aromatic-ring-connecting C—C bond and a weak C—H⋯π inter­action (2.86 and 2.99 Å) between the two aromatic rings.

## Related literature

For related literature, see: Dougherty *et al.* (1978 ▶); Graebe & Manz (1896 ▶); Olah *et al.* (1981 ▶); Solans *et al.* (1980 ▶); Sridevi *et al.* (2006 ▶).
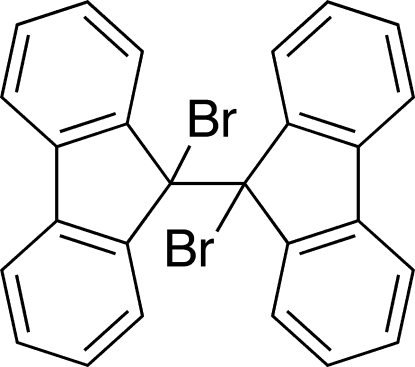


## Experimental

### 

#### Crystal data

C_26_H_16_Br_2_
*M* *_r_* = 488.21
Monoclinic, 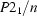

*a* = 12.7083 (3) Å
*b* = 12.0480 (2) Å
*c* = 12.7786 (2) Å
β = 102.5340 (8)°
*V* = 1909.90 (6) Å^3^
*Z* = 4
Mo *K*α radiation
μ = 4.25 mm^−1^
*T* = 103 (2) K
0.30 × 0.10 × 0.10 mm

#### Data collection

Rigaku Mercury CCD diffractometer
Absorption correction: multi-scan (*REQUAB*; Jacobson, 1998 ▶) *T*_min_ = 0.351, *T*_max_ = 0.655
12332 measured reflections
3339 independent reflections
3242 reflections with *I* > 2σ(*I*)
*R*_int_ = 0.039

#### Refinement

*R*[*F* ^2^ > 2σ(*F* ^2^)] = 0.027
*wR*(*F* ^2^) = 0.070
*S* = 1.12
3339 reflections
254 parameters
H-atom parameters constrained
Δρ_max_ = 0.48 e Å^−3^
Δρ_min_ = −0.97 e Å^−3^

### 

Data collection: *CrystalClear* (Rigaku, 2004 ▶); cell refinement: *CrystalClear*; data reduction: *CrystalClear*; program(s) used to solve structure: *SHELXS97* (Sheldrick, 2008 ▶); program(s) used to refine structure: *SHELXL97* (Sheldrick, 2008 ▶); molecular graphics: *ORTEPIII* (Burnett & Johnson, 1996 ▶) and *Mercury* (Version 1.4.2; Macrae *et al.*, 2006 ▶); software used to prepare material for publication: *yadokari-XG* (Wakita, 2005 ▶).

## Supplementary Material

Crystal structure: contains datablocks global, I. DOI: 10.1107/S160053680800295X/sg2222sup1.cif

Structure factors: contains datablocks I. DOI: 10.1107/S160053680800295X/sg2222Isup2.hkl

Additional supplementary materials:  crystallographic information; 3D view; checkCIF report

## Figures and Tables

**Table 1:** Selected interatomic distances (Å)

Br2⋯C11^i^	3.299 (2)
Br2⋯C12^i^	3.369 (2)

Symmetry code: (i) 
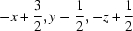
.

**Table 2:** Hydrogen-bond geometry (Å, °)

*D*—H⋯*A*	*D*—H	H⋯*A*	*D*⋯*A*	*D*—H⋯*A*
C20—H15⋯C2^ii^	0.95	2.99	3.760 (3)	140
C20—H15⋯C3^ii^	0.95	2.86	3.493 (3)	125

Symmetry code: (ii) 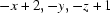.

**Fig. 1.**
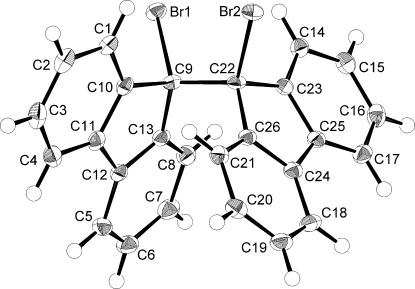
The molecular structure of the title compound, showing the atom-labeling scheme. Displacement ellipsoids are drawn at the 50% probability level.

**Fig. 2.**
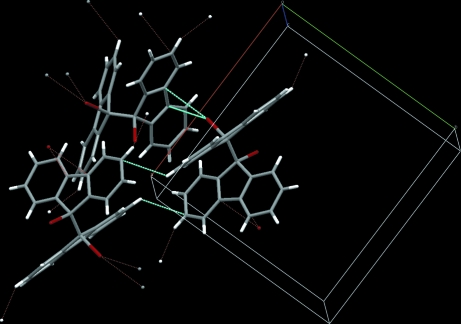
The molecular packing of the title compound. Dashed lines indicate the intermolecular contacts [symmetry codes: (i) 3/2 - x, -1/2 + y, 1/2 - z, (ii) 2 - x, -y, 1 - z].

## supplementary crystallographic information

### Comment

Although a number of synthetic works and structural analyses on 9,9'-bifluorene derivertives have been reported, only one report is known for the crystalline structure of 9-halo-9,9'-bifluorene derivertive, that is, 1,1',2,2',3,3',4,4',5,5',6,6',7,7',8,8',9-heptadecachloro- 9,9'-bifluorene (Solans *et al.*, 1980). We have succeeded in the first structural analysis of 9,9'-dihalo-9,9'-bifluorene derivertive, 9,9'-dibromo-9,9'-bifluorene.

The first report on the synthesis of 9,9'-dibromo-9,9'-bifluorene appaered in the 19t h century (Graebe *et al.*, 1896). Its *gauche* groud-state conformation was revealed by its NMR spectra and the theoretical calculations (Olah *et al.*, 1981).

The molecular structure of the title compound is shown in Fig. 1. The dihedral angle of Br1—C9—C22—Br2 is 59.39 (16)°, indicating its *gauche* conformation. Although the bond length of C9—C22 [1.561 (3) Å] is longer than that of non-substituted 9,9'-bifluorene [1.542 (3) Å, Dougherty *et al.*, 1978; 1.539 (3) Å, Sridevi *et al.*, 2006], it is one of the shortest C—C bonds between 9 and 9' positions among the 9(,9')-substituted 9,9'-bifluorenes (1.559–1.724 Å). The shortest intermolecular contacts were found to be C22—Br2···C11^i^ [3.299 (2) Å], C22—Br2···C12^i^ [3.369 (2) Å], and C20—H15···C3^ii^ (2.86 Å) (Fig. 2) [symmetry codes: (i) 3/2 - *x*, -1/2 + *y*, 1/2 - *z*, (ii) 2 - *x*, -*y*, 1 - *z*].

### Refinement

H atoms were placed in geometrically idealized positions and allowed to ride on their parent atoms with C—H = 0.95 Å and *U*_iso_(H) = 1.2*U*_eq_(C).

### Crystal data

**Table:**

C_26_H_16_Br_2_	*Z* = 4
*M**_r_* = 488.21	*F*_000_ = 968
Monoclinic, *P*2_1_/*n*	*D*_x_ = 1.698 Mg m^−^^3^
Hall symbol: -P 2yn	Mo *K*α radiation λ = 0.71069 Å
*a* = 12.7083 (3) Å	θ = 3.1–25.0º
*b* = 12.0480 (2) Å	µ = 4.25 mm^−^^1^
*c* = 12.7786 (2) Å	*T* = 103 (2) K
β = 102.5340 (8)º	Prism, colorless
*V* = 1909.90 (6) Å^3^	0.30 × 0.10 × 0.10 mm

### Data collection

**Table:**

Rigaku Mercury CCD diffractometer	3339 independent reflections
Radiation source: fine-focus sealed tube	3242 reflections with *I* > 2σ(*I*)
Monochromator: graphite	*R*_int_ = 0.039
*T* = 103(2) K	θ_max_ = 25.0º
ω scans	θ_min_ = 3.1º
Absorption correction: multi-scan(REQUAB; Jacobson, 1998)	*h* = −15→11
*T*_min_ = 0.351, *T*_max_ = 0.655	*k* = −14→14
12332 measured reflections	*l* = −11→15

### Refinement

**Table:**

Refinement on *F*^2^	Hydrogen site location: inferred from neighbouring sites
Least-squares matrix: full	H-atom parameters constrained
*R*[*F*^2^ > 2σ(*F*^2^)] = 0.027	*w* = 1/[σ^2^(*F*_o_^2^) + (0.0444*P*)^2^ + 0.8804*P*] where *P* = (*F*_o_^2^ + 2*F*_c_^2^)/3
*wR*(*F*^2^) = 0.070	(Δ/σ)_max_ = 0.001
*S* = 1.12	Δρ_max_ = 0.48 e Å^−^^3^
3339 reflections	Δρ_min_ = −0.97 e Å^−^^3^
254 parameters	Extinction correction: SHELXL97 (Sheldrick, 2008), Fc^*^=kFc[1+0.001xFc^2^λ^3^/sin(2θ)]^-1/4^
Primary atom site location: structure-invariant direct methods	Extinction coefficient: 0.0188 (9)
Secondary atom site location: difference Fourier map	

### Special details

**Table:**

Geometry. All e.s.d.'s (except the e.s.d. in the dihedral angle between two l.s. planes) are estimated using the full covariance matrix. The cell e.s.d.'s are taken into account individually in the estimation of e.s.d.'s in distances, angles and torsion angles; correlations between e.s.d.'s in cell parameters are only used when they are defined by crystal symmetry. An approximate (isotropic) treatment of cell e.s.d.'s is used for estimating e.s.d.'s involving l.s. planes.
Refinement. Refinement of *F*^2^ against ALL reflections. The weighted *R*-factor *wR* and goodness of fit *S* are based on *F*^2^, conventional *R*-factors *R* are based on *F*, with *F* set to zero for negative *F*^2^. The threshold expression of *F*^2^ > σ(*F*^2^) is used only for calculating *R*-factors(gt) *etc*. and is not relevant to the choice of reflections for refinement. *R*-factors based on *F*^2^ are statistically about twice as large as those based on *F*, and *R*- factors based on ALL data will be even larger.

### Fractional atomic coordinates and isotropic or equivalent isotropic displacement parameters (Å^2^)

**Table:**

	*x*	*y*	*z*	*U*_iso_*/*U*_eq_	
C1	0.84656 (18)	0.16573 (18)	0.23137 (17)	0.0212 (4)	
H1	0.7992	0.1271	0.1755	0.025*	
C2	0.95781 (19)	0.15777 (19)	0.24391 (19)	0.0253 (5)	
H2	0.9867	0.1129	0.1959	0.030*	
C3	1.02751 (18)	0.21444 (19)	0.3257 (2)	0.0261 (5)	
H3	1.1032	0.2067	0.3332	0.031*	
C4	0.98803 (18)	0.28206 (18)	0.39616 (18)	0.0227 (5)	
H4	1.0356	0.3213	0.4514	0.027*	
C5	0.84633 (19)	0.43653 (19)	0.52326 (17)	0.0241 (5)	
H5	0.9206	0.4459	0.5551	0.029*	
C6	0.7676 (2)	0.4993 (2)	0.55742 (18)	0.0270 (5)	
H6	0.7885	0.5524	0.6130	0.032*	
C7	0.6587 (2)	0.48479 (18)	0.51094 (18)	0.0254 (5)	
H7	0.6063	0.5289	0.5345	0.031*	
C8	0.62552 (18)	0.40646 (17)	0.43032 (17)	0.0214 (4)	
H8	0.5512	0.3961	0.3993	0.026*	
C9	0.69082 (16)	0.25349 (16)	0.31151 (16)	0.0165 (4)	
C10	0.80673 (17)	0.23199 (16)	0.30326 (16)	0.0174 (4)	
C11	0.87685 (17)	0.29089 (17)	0.38378 (17)	0.0195 (4)	
C12	0.81287 (17)	0.36011 (17)	0.44157 (16)	0.0189 (4)	
C13	0.70351 (18)	0.34400 (17)	0.39632 (16)	0.0180 (4)	
C14	0.42727 (17)	0.20938 (17)	0.30227 (18)	0.0196 (4)	
H9	0.4246	0.2216	0.2283	0.024*	
C15	0.33643 (18)	0.22638 (17)	0.34502 (19)	0.0223 (5)	
H10	0.2715	0.2515	0.2995	0.027*	
C16	0.33917 (18)	0.20728 (18)	0.45294 (19)	0.0237 (5)	
H11	0.2765	0.2204	0.4804	0.028*	
C17	0.43250 (19)	0.16928 (17)	0.52080 (18)	0.0212 (4)	
H12	0.4340	0.1546	0.5942	0.025*	
C18	0.67213 (19)	0.07827 (17)	0.63627 (17)	0.0224 (5)	
H13	0.6286	0.0808	0.6880	0.027*	
C19	0.7776 (2)	0.04043 (17)	0.66387 (19)	0.0241 (5)	
H14	0.8070	0.0184	0.7356	0.029*	
C20	0.84104 (19)	0.03434 (17)	0.58756 (18)	0.0223 (5)	
H15	0.9131	0.0081	0.6080	0.027*	
C21	0.79998 (17)	0.06636 (17)	0.48168 (17)	0.0195 (4)	
H16	0.8424	0.0603	0.4292	0.023*	
C22	0.63207 (16)	0.15126 (16)	0.34754 (15)	0.0161 (4)	
C23	0.52149 (17)	0.17426 (16)	0.37060 (16)	0.0167 (4)	
C24	0.52399 (17)	0.15302 (16)	0.47932 (16)	0.0178 (4)	
C25	0.63132 (17)	0.11255 (16)	0.53100 (16)	0.0182 (4)	
C26	0.69575 (17)	0.10723 (16)	0.45519 (16)	0.0169 (4)	
Br1	0.611538 (16)	0.312530 (17)	0.171820 (15)	0.02031 (11)	
Br2	0.616445 (16)	0.029301 (16)	0.241602 (16)	0.02030 (11)	

### Atomic displacement parameters (Å^2^)

**Table:**

	*U*^11^	*U*^22^	*U*^33^	*U*^12^	*U*^13^	*U*^23^
C1	0.0244 (11)	0.0232 (10)	0.0175 (10)	0.0005 (8)	0.0074 (9)	0.0037 (9)
C2	0.0270 (12)	0.0252 (11)	0.0271 (12)	0.0070 (9)	0.0131 (10)	0.0082 (10)
C3	0.0188 (11)	0.0271 (11)	0.0334 (13)	0.0031 (9)	0.0082 (10)	0.0119 (10)
C4	0.0196 (11)	0.0224 (10)	0.0242 (11)	−0.0016 (8)	0.0003 (9)	0.0085 (9)
C5	0.0277 (12)	0.0251 (11)	0.0170 (11)	−0.0068 (9)	−0.0005 (9)	0.0024 (9)
C6	0.0407 (14)	0.0228 (11)	0.0182 (11)	−0.0077 (10)	0.0080 (10)	−0.0052 (9)
C7	0.0356 (13)	0.0215 (11)	0.0217 (11)	−0.0012 (9)	0.0117 (10)	−0.0037 (9)
C8	0.0253 (11)	0.0184 (10)	0.0214 (11)	−0.0017 (8)	0.0068 (9)	−0.0005 (8)
C9	0.0195 (10)	0.0178 (9)	0.0114 (9)	0.0013 (8)	0.0021 (8)	0.0004 (8)
C10	0.0195 (10)	0.0175 (10)	0.0153 (10)	0.0016 (8)	0.0044 (8)	0.0056 (8)
C11	0.0222 (11)	0.0183 (10)	0.0177 (10)	−0.0001 (8)	0.0040 (9)	0.0081 (8)
C12	0.0245 (11)	0.0178 (10)	0.0143 (10)	−0.0026 (8)	0.0037 (8)	0.0043 (8)
C13	0.0249 (11)	0.0157 (9)	0.0135 (10)	−0.0019 (8)	0.0046 (8)	0.0023 (8)
C14	0.0215 (11)	0.0179 (10)	0.0198 (11)	−0.0017 (8)	0.0051 (9)	0.0000 (8)
C15	0.0192 (11)	0.0194 (10)	0.0275 (12)	0.0002 (8)	0.0034 (9)	−0.0002 (9)
C16	0.0236 (11)	0.0199 (10)	0.0303 (12)	−0.0014 (8)	0.0116 (10)	−0.0023 (9)
C17	0.0288 (12)	0.0175 (10)	0.0190 (11)	−0.0024 (8)	0.0088 (9)	−0.0018 (8)
C18	0.0330 (12)	0.0196 (10)	0.0146 (10)	−0.0012 (9)	0.0049 (9)	−0.0005 (8)
C19	0.0323 (13)	0.0210 (10)	0.0162 (11)	−0.0012 (9)	−0.0008 (9)	0.0021 (8)
C20	0.0240 (12)	0.0174 (10)	0.0221 (11)	0.0009 (8)	−0.0021 (9)	0.0031 (8)
C21	0.0225 (11)	0.0153 (10)	0.0203 (11)	−0.0006 (8)	0.0035 (8)	0.0007 (8)
C22	0.0205 (11)	0.0154 (9)	0.0118 (9)	−0.0003 (8)	0.0023 (8)	−0.0037 (8)
C23	0.0196 (11)	0.0143 (9)	0.0164 (10)	−0.0027 (7)	0.0044 (8)	−0.0031 (8)
C24	0.0231 (11)	0.0136 (9)	0.0167 (10)	−0.0027 (8)	0.0044 (8)	−0.0032 (8)
C25	0.0249 (11)	0.0125 (9)	0.0167 (10)	−0.0021 (8)	0.0035 (9)	−0.0021 (8)
C26	0.0229 (10)	0.0132 (9)	0.0141 (10)	−0.0021 (8)	0.0026 (8)	−0.0007 (7)
Br1	0.02204 (15)	0.02365 (15)	0.01439 (15)	0.00219 (7)	0.00212 (9)	0.00367 (7)
Br2	0.02386 (16)	0.01924 (15)	0.01694 (14)	0.00053 (7)	0.00257 (9)	−0.00527 (7)

### Geometric parameters (Å, °)

**Table:**

C1—C2	1.391 (3)	C14—C23	1.386 (3)
C1—C10	1.393 (3)	C14—C15	1.395 (3)
C1—H1	0.9500	C14—H9	0.9500
C2—C3	1.393 (4)	C15—C16	1.391 (3)
C2—H2	0.9500	C15—H10	0.9500
C3—C4	1.387 (3)	C16—C17	1.386 (3)
C3—H3	0.9500	C16—H11	0.9500
C4—C11	1.391 (3)	C17—C24	1.392 (3)
C4—H4	0.9500	C17—H12	0.9500
C5—C12	1.388 (3)	C18—C19	1.387 (3)
C5—C6	1.398 (4)	C18—C25	1.395 (3)
C5—H5	0.9500	C18—H13	0.9500
C6—C7	1.394 (4)	C19—C20	1.395 (3)
C6—H6	0.9500	C19—H14	0.9500
C7—C8	1.393 (3)	C20—C21	1.395 (3)
C7—H7	0.9500	C20—H15	0.9500
C8—C13	1.387 (3)	C21—C26	1.385 (3)
C8—H8	0.9500	C21—H16	0.9500
C9—C13	1.521 (3)	C22—C23	1.523 (3)
C9—C10	1.522 (3)	C22—C26	1.531 (3)
C9—C22	1.561 (3)	C22—Br2	1.9785 (19)
C9—Br1	1.982 (2)	C23—C24	1.406 (3)
C10—C11	1.399 (3)	C24—C25	1.464 (3)
C11—C12	1.470 (3)	C25—C26	1.399 (3)
C12—C13	1.398 (3)		
			
Br2···C11^i^	3.299 (2)	Br2···C12^i^	3.369 (2)
			
C2—C1—C10	118.0 (2)	C23—C14—C15	118.2 (2)
C2—C1—H1	121.0	C23—C14—H9	120.9
C10—C1—H1	121.0	C15—C14—H9	120.9
C1—C2—C3	121.2 (2)	C16—C15—C14	121.3 (2)
C1—C2—H2	119.4	C16—C15—H10	119.4
C3—C2—H2	119.4	C14—C15—H10	119.4
C4—C3—C2	120.9 (2)	C17—C16—C15	120.6 (2)
C4—C3—H3	119.5	C17—C16—H11	119.7
C2—C3—H3	119.5	C15—C16—H11	119.7
C3—C4—C11	118.2 (2)	C16—C17—C24	118.7 (2)
C3—C4—H4	120.9	C16—C17—H12	120.7
C11—C4—H4	120.9	C24—C17—H12	120.7
C12—C5—C6	118.1 (2)	C19—C18—C25	118.6 (2)
C12—C5—H5	121.0	C19—C18—H13	120.7
C6—C5—H5	121.0	C25—C18—H13	120.7
C7—C6—C5	120.8 (2)	C18—C19—C20	120.8 (2)
C7—C6—H6	119.6	C18—C19—H14	119.6
C5—C6—H6	119.6	C20—C19—H14	119.6
C8—C7—C6	120.9 (2)	C21—C20—C19	120.8 (2)
C8—C7—H7	119.6	C21—C20—H15	119.6
C6—C7—H7	119.6	C19—C20—H15	119.6
C13—C8—C7	118.5 (2)	C26—C21—C20	118.2 (2)
C13—C8—H8	120.8	C26—C21—H16	120.9
C7—C8—H8	120.8	C20—C21—H16	120.9
C13—C9—C10	102.52 (16)	C23—C22—C26	102.66 (16)
C13—C9—C22	109.82 (16)	C23—C22—C9	115.74 (16)
C10—C9—C22	114.74 (16)	C26—C22—C9	110.18 (16)
C13—C9—Br1	109.82 (13)	C23—C22—Br2	107.92 (13)
C10—C9—Br1	108.01 (13)	C26—C22—Br2	108.20 (13)
C22—C9—Br1	111.52 (13)	C9—C22—Br2	111.57 (13)
C1—C10—C11	120.7 (2)	C14—C23—C24	120.61 (19)
C1—C10—C9	129.73 (19)	C14—C23—C22	129.87 (19)
C11—C10—C9	109.53 (18)	C24—C23—C22	109.52 (18)
C4—C11—C10	120.9 (2)	C17—C24—C23	120.6 (2)
C4—C11—C12	130.1 (2)	C17—C24—C25	130.42 (19)
C10—C11—C12	108.88 (18)	C23—C24—C25	108.98 (18)
C5—C12—C13	121.2 (2)	C18—C25—C26	120.2 (2)
C5—C12—C11	129.9 (2)	C18—C25—C24	130.43 (19)
C13—C12—C11	108.78 (18)	C26—C25—C24	109.33 (18)
C8—C13—C12	120.6 (2)	C21—C26—C25	121.31 (19)
C8—C13—C9	129.8 (2)	C21—C26—C22	129.33 (18)
C12—C13—C9	109.65 (18)	C25—C26—C22	109.36 (17)
			
C10—C1—C2—C3	−0.1 (3)	C19—C20—C21—C26	1.8 (3)
C1—C2—C3—C4	−1.0 (3)	C13—C9—C22—C23	57.5 (2)
C2—C3—C4—C11	0.7 (3)	C10—C9—C22—C23	172.27 (17)
C12—C5—C6—C7	0.3 (3)	Br1—C9—C22—C23	−64.52 (19)
C5—C6—C7—C8	0.9 (3)	C13—C9—C22—C26	−58.4 (2)
C6—C7—C8—C13	−0.8 (3)	C10—C9—C22—C26	56.4 (2)
C2—C1—C10—C11	1.6 (3)	Br1—C9—C22—C26	179.60 (13)
C2—C1—C10—C9	−177.91 (19)	C13—C9—C22—Br2	−178.64 (13)
C13—C9—C10—C1	−172.8 (2)	C10—C9—C22—Br2	−63.81 (19)
C22—C9—C10—C1	68.2 (3)	Br1—C9—C22—Br2	59.39 (16)
Br1—C9—C10—C1	−56.9 (2)	C15—C14—C23—C24	2.0 (3)
C13—C9—C10—C11	7.6 (2)	C15—C14—C23—C22	−178.76 (19)
C22—C9—C10—C11	−111.41 (19)	C26—C22—C23—C14	−175.8 (2)
Br1—C9—C10—C11	123.53 (15)	C9—C22—C23—C14	64.1 (3)
C3—C4—C11—C10	0.8 (3)	Br2—C22—C23—C14	−61.7 (2)
C3—C4—C11—C12	−176.1 (2)	C26—C22—C23—C24	3.5 (2)
C1—C10—C11—C4	−2.0 (3)	C9—C22—C23—C24	−116.61 (19)
C9—C10—C11—C4	177.61 (18)	Br2—C22—C23—C24	117.60 (15)
C1—C10—C11—C12	175.47 (18)	C16—C17—C24—C23	−0.3 (3)
C9—C10—C11—C12	−4.9 (2)	C16—C17—C24—C25	−178.9 (2)
C6—C5—C12—C13	−1.6 (3)	C14—C23—C24—C17	−1.4 (3)
C6—C5—C12—C11	174.0 (2)	C22—C23—C24—C17	179.19 (18)
C4—C11—C12—C5	1.0 (4)	C14—C23—C24—C25	177.42 (17)
C10—C11—C12—C5	−176.2 (2)	C22—C23—C24—C25	−1.9 (2)
C4—C11—C12—C13	177.0 (2)	C19—C18—C25—C26	0.8 (3)
C10—C11—C12—C13	−0.2 (2)	C19—C18—C25—C24	178.3 (2)
C7—C8—C13—C12	−0.5 (3)	C17—C24—C25—C18	0.4 (4)
C7—C8—C13—C9	179.6 (2)	C23—C24—C25—C18	−178.3 (2)
C5—C12—C13—C8	1.7 (3)	C17—C24—C25—C26	178.1 (2)
C11—C12—C13—C8	−174.74 (18)	C23—C24—C25—C26	−0.6 (2)
C5—C12—C13—C9	−178.35 (18)	C20—C21—C26—C25	−2.5 (3)
C11—C12—C13—C9	5.2 (2)	C20—C21—C26—C22	177.94 (19)
C10—C9—C13—C8	172.2 (2)	C18—C25—C26—C21	1.2 (3)
C22—C9—C13—C8	−65.4 (3)	C24—C25—C26—C21	−176.82 (18)
Br1—C9—C13—C8	57.6 (3)	C18—C25—C26—C22	−179.13 (17)
C10—C9—C13—C12	−7.7 (2)	C24—C25—C26—C22	2.9 (2)
C22—C9—C13—C12	114.67 (19)	C23—C22—C26—C21	175.8 (2)
Br1—C9—C13—C12	−122.35 (15)	C9—C22—C26—C21	−60.3 (3)
C23—C14—C15—C16	−0.9 (3)	Br2—C22—C26—C21	61.9 (2)
C14—C15—C16—C17	−0.8 (3)	C23—C22—C26—C25	−3.8 (2)
C15—C16—C17—C24	1.4 (3)	C9—C22—C26—C25	120.03 (18)
C25—C18—C19—C20	−1.4 (3)	Br2—C22—C26—C25	−117.75 (15)
C18—C19—C20—C21	0.1 (3)		

Symmetry codes: (i) −*x*+3/2, *y*−1/2, −*z*+1/2.

### Hydrogen-bond geometry (Å, °)

**Table:**

*D*—H···*A*	*D*—H	H···*A*	*D*···*A*	*D*—H···*A*
C20—H15···C2^ii^	0.95	2.99	3.760 (3)	140
C20—H15···C3^ii^	0.95	2.86	3.493 (3)	125

Symmetry codes: (ii) −*x*+2, −*y*, −*z*+1.
